# Genome-Wide Identification of the *IQM* Gene Family and Their Transcriptional Responses to Abiotic Stresses in Kiwifruit (*Actinidia eriantha*)

**DOI:** 10.3390/genes15020147

**Published:** 2024-01-23

**Authors:** Minyan Xu, Zhi Zhang, Chengcheng Ling, Yuhuan Jiao, Xin Zhang

**Affiliations:** 1 National Engineering Laboratory of Crop Stress Resistance Breeding, School of Life Sciences, Anhui Agricultural University, Hefei 230036, China; 2 College of Food and Bioengineering, Bengbu University, Bengbu 233030, China

**Keywords:** calcium-binding protein, *AeIQM*, salt, hormone, abiotic stress

## Abstract

IQM is a plant-specific calcium-binding protein that plays a pivotal role in various aspects of plant growth response to stressors. We investigated the *IQM* gene family and its expression patterns under diverse abiotic stresses and conducted a comprehensive analysis and characterization of the *AeIQMs*, including protein structure, genomic location, phylogenetic relationships, gene expression profiles, salt tolerance, and expression patterns of this gene family under different abiotic stresses. Based on phylogenetic analysis, these 10 AeIQMs were classified into three distinct subfamilies (I–III). Analysis of the protein motifs revealed a considerable level of conservation among these AeIQM proteins within their respective subfamilies in kiwifruit. The genomic distribution of the 10 *AeIQM* genes spanned across eight chromosomes, where four pairs of *IQM* gene duplicates were associated with segmental duplication events. qRT-PCR analysis revealed diverse expression patterns of these *AeIQM* genes under different hormone treatments, and most *AeIQMs* showed inducibility by salt stress. Further investigations indicated that overexpression of *AeIQMs* in yeast significantly enhanced salt tolerance. These findings suggest that *AeIQM* genes might be involved in hormonal signal transduction and response to abiotic stress in *Actinidia eriantha*. In summary, this study provides valuable insights into the physiological functions of *IQMs* in kiwifruit.

## 1. Introduction

Various stresses influence plant growth and development [1]. Notably, abiotic stressors, including high temperature, salinity, drought, and cold, are widely recognized as primary factors contributing to global crop yield reduction [2,3,4,5]. Soil salinization, in particular, is a widespread problem, altering soil characteristics, reducing moisture content, and compacting the soil, resulting in hindered plant growth and development [2]. Unlike animals, plants cannot relocate to avoid unfavorable conditions. However, during the process of evolution, they have developed special adaptation mechanisms to cope with these rapidly changing stressors [6,7,8]. Many signaling pathways are involved in plants’ responses to abiotic stresses. Among them, calcium ions (Ca^2+^) play a crucial role as an important second messenger in plants’ adaptation to external stimuli [9,10,11].

Plants primarily monitor spatial and temporal changes in cytosolic Ca^2+^ levels through specific Ca^2+^ sensors and convert these Ca^2+^ level changes into a series of downstream effects [12]. Ca^2+^ sensors are divided into four classes based on the number of Ca^2+^-binding helix–loop–helix EF-hand motifs they contain, such as calmodulins (CaMs), calcineurin B-like proteins (CBLs), calcium-dependent protein kinases (CDPKs), and calmodulin-like proteins (CMLs) [9,13,14]. Among them, calmodulin is a highly conserved, small, acidic protein. CaM itself lacks enzymatic activity but undergoes conformational changes upon Ca^2+^ binding, thereby activating downstream target proteins, including kinases, metabolic enzymes, cytoskeletal proteins, and transcription factors, and participating in various cellular processes [9,12,15]. CaM-binding proteins can be categorized into two types: Ca^2+^-dependent and Ca^2+^-independent. Ca^2+^-independent types include CaM-binding proteins containing IQ motifs. In plants, based on differences in structural domains and the number of IQ motifs present, these CaM-binding proteins are classified into five families: the IQ motif-containing protein (IQM) [16], IQ67-domain-containing protein (IQD) [17], cyclic nucleotide-gated channel (CNGC) [18], calmodulin-binding transcription activator (CAMTA) [19], and myosin families [20].

The *IQM* family is a very important class of calmodulin-binding protein family genes, which are widely present in various organs of plants [16,21,22]. IQM contains a single IQ motif that binds to calmodulin, located at the N-terminus of the amino acid sequence [16]. Previous research has demonstrated marked variations in the expression profiles of *IQMs* across diverse tissues and responses to various stress conditions. These dynamic expression patterns denote their potential involvement in various physiological regulatory processes associated with plant growth, development, and stress responses. The *IQM* gene family has been successfully identified in some plant species, including *Arabidopsis* [23], soybean [21], and rice [22]. *Arabidopsis* has six *IQM* gene family members (*AtIQM1*-*AtIQM6*), with *IQM1* being involved in regulating stomatal movement, root growth, jasmonic acid methyl ester biosynthesis, and defense against gray mold [23,24]. *IQM4* plays a role in seed dormancy, germination, and growth [25]. Mutations in *IQM5* can result in altered flowering time phenotypes [26]. Eight *OsIQM* gene family members have been reported in rice, and studies have shown that OsIQMs participate in regulating various pathways through interaction with IQ motifs in OsCaMs. Additionally, almost all *OsIQMs* genes are responsive to ABA and MeJA [22].

However, despite notable progress in the identification and characterization of *IQM* gene family members across some plant species, comprehensive knowledge regarding their presence and structural attributes in kiwifruit remains limited. Therefore, the present study endeavors to conduct an extensive analysis of the *IQM* gene family in kiwifruit. This investigation encompasses genome-wide scale methodologies, as well as molecular biology techniques, to unravel essential aspects such as phylogenetic relationships, conserved motifs, chromosome localization, gene structure, and expression profiles in response to both abiotic stressors and hormonal stimuli within the *AeIQM* family. Moreover, kiwifruit is highly susceptible to changes in soil salt content, which can disrupt cellular ion balance, hinder enzyme functions, and hamper metabolic networks, consequently affecting cell growth and development [27,28,29,30]. Prior studies have demonstrated that kiwifruit growth and development are adversely affected even by soil salt contents as low as 0.14%, resulting in diminished accumulation of organic matter and shorter internode lengths. Furthermore, a salt content of 0.54% exerts a further deleterious impact, leading to reduced yield and lower tree survival rates [31]. Building upon these findings, we conducted a subsequent investigation focusing on examining the expression levels of the *IQM* gene family under salt stress conditions. Additionally, we employed the strategy of overexpressing *IQM* genes in yeast to ascertain their responsiveness to salt stress. By providing significant theoretical insights into the structural characteristics and potential functionalities of *IQM* genes in kiwifruit, this study holds exceptional importance for advancing our understanding and enhancing kiwifruit’s ability to flourish in salt-stress environments.

## 2. Materials and Methods

### 2.1. Identification of Kiwifruit IQM Proteins

The *A. eriantha* (White) genome sequences were downloaded from the Kiwifruit Genome Database (https://kiwifruitgenome.org/, accessed on 17 November 2023). The *Arabidopsis* and *Oryza sativa* IQM protein sequences were retrieved from the Phytozome v13 database (http://phytozome-next.jgi.doe.gov, accessed on 17 November 2023) [32]. The six AtIQM and eight OsIQM protein sequences were used as query sequences to search against the kiwifruit protein databases using the BLASTP program, with an e-value of 1 × e^−50^ as the threshold. The conserved domains of candidate sequences were annotated using the SMART (http://smart.emblheidelberg.de/, accessed on 20 November 2023) [33] and Pfamscan (https://www.ebi.ac.uk/Tools/pfa/pfamscan/, accessed on 20 November 2023) databases [34]. Members of the kiwifruit IQM family were identified based on the presence of the complete IQ motif. Information on *AeIQM* genes, including genome sequences, location coordinates, ORF lengths, amino acid numbers, and molecular weight, were obtained from the kiwifruit genome database. The physicochemical characteristics of the AeIQMs were generated via ExPASy (http://web.expasy.org/protparam/, accessed on 20 November 2023), a comprehensive resource for protein analysis [35].

### 2.2. Phylogenetic Analysis

To investigate the evolutionary relationships of AeIQMs within and between species, we obtained predicted IQM protein sequences from corresponding databases for multiple species. Specifically, we retrieved IQM protein sequences from kiwifruit (*A. eriantha*, White), rice (*Oryza sativa*-IRGSP-1.0), and *Arabidopsis thaliana* from the kiwifruit genome database and Phytozome v13 database. The phylogenetic tree was constructed using the neighbor-joining (NJ) method via MEGA software (v11), applying the P-distance substitution model. Bootstrap analysis was performed using 1000 replicates [36]. The generated phylogenetic tree was visualized using Chiplot (https://www.chiplot.online/, accessed on 20 December 2023).

### 2.3. Chromosomal Localization and Gene Duplication

The TBtools software (v2.027) was utilized to determine the chromosomal distribution and gene duplication events of *AeIQM* genes [37] based on their positional information obtained from the kiwifruit genome database. To investigate the evolutionary divergence of duplicated genes, the non-synonymous substitution rate (Ka) and synonymous substitution rate (Ks) were computed using the TBtools software. To establish the evolutionary relationships, the genome databases and gene annotation files of kiwifruit were subjected to analysis using the MCScanX function, which facilitated the identification of homologous gene pairs [38]. Subsequently, the Ka/Ks function was employed for the purpose of calculating the evolutionary divergence.

### 2.4. Analysis of the Gene Structures, Conserved Motifs, and Cis-Acting Elements of AeIQM

To analyze the structure of *AeIQM* genes, we downloaded the coding sequence (CDS) and gff3 format files of *AeIQM* genes from the kiwifruit genome database. Subsequently, we utilized TBtools software to analyze the exon/intron structure for each *IQM* gene. To identify conserved motifs within AeIQM proteins, we utilized Multiple Expectation Maximization for Motif Elicitation (MEME, http://meme.nbcr.net/meme/cgibin/meme.cgi, accessed on 20 November 2023) to analyze the complete encoded AeIQM amino acid sequences [39], setting the maximum number of motifs as 15. Additionally, we obtained the 2 kbp promoter sequences of *AeIQM* genes from the kiwifruit genome database. Subsequently, the obtained sequences were subjected to analysis using the PlantCARE Database website (https://bioinformatics.psb.ugent.be/webtools/plantcare/html/, accessed on 27 November 2023) to predict the presence of *cis*-acting elements. The obtained results were visualized using TBtools software (v2.027) [37].

### 2.5. Plant Materials’ Growth Conditions, Hormones, and Abiotic Treatments

The kiwifruit seedlings were cultured in a soil mixture of perlite and sand (3:1, *v*/*v*) under specific conditions of 24 °C, 14 h light and 10 h dark, and a relative humidity of 60–80%. To evaluate the changes in the expression levels of *AeIQM* genes in response to diverse hormone and abiotic stress stimuli, seedlings (plant height: 25–30 cm) were randomly divided into six groups for stress treatments. For hormone treatments, after acquiring each hormone from Sigma (Sigma-Aldrich, St. Louis, MO, USA), aqueous solutions of 300 μM abscisic acid (ABA), 100 μM gibberellin (GA), 100 μM salicylic acid (SA), and 50 μM methyl jasmonate (MeJA) were sprayed onto the leaves of the kiwifruit (both sides of the leaves), respectively [7]. The treated seedlings were harvested at various time points following the treatment, including at 0, 6, 12, 24, and 48 h. Regarding the application of salt and drought stresses, plants 25–30 cm in height were immersed in 0.6% NaCl solution for 6 consecutive days, and the drought stress (direct drought treatment by stopping water supply) involved drying the seedlings for 14 days [40]. The control group consisted of untreated seedlings (CK).

### 2.6. RNA Isolation, Quantitative Real-Time PCR, and Heatmap

Total RNA was extracted from various kiwifruit samples (samples taken before and after each hormone/stress treatment) using the RNA Midi Kit (OMEGA, Guangzhou, China), following the manufacturer’s protocols. RNA extraction was conducted in triplicate using three biological replicates. Subsequently, reverse transcription was carried out using the First-Strand cDNA Synthesis Kit (Vazyme, Nanjing, China). For quantitative real-time PCR (qRT-PCR), the Thermo Scientific Pikoreal Cycler 96 Real-Time PCR system was employed with SYBR Green Master Mix (Vazyme, Nanjing, China). The internal control for mRNA was *AeActin1*, and the relative expression levels of genes were determined using the 2^−∆∆CT^ method [41]. All experiments were performed in triplicate. Detailed information about the primer sequences can be found in Supplementary Materials Table S1. A heatmap was generated from real-time quantitative PCR analysis of *AeIQM* genes after hormone treatment. Values are the mean of three technical measurements. TBtools software (v2.027) normalized the data to obtain relative intensity values, with red indicating high expression levels and blue indicating low expression levels [38].

### 2.7. Salt Stress Tolerance Assays of IQM Genes in Yeast

The open reading frame (ORF) of *IQM* genes was amplified from the *A. eriantha* cDNA library through the utilization of primers designed with *BamH*I and *EcoR*I sites incorporated. The PCR-amplified products were then digested and inserted into the corresponding sites of the yeast expression vector pYES2-NT B, which harbors the URA3 selection marker controlled by a GAL1 promoter. The resulting recombinant plasmids (pYES2-AeIQMs) were separately transformed into *Saccharomyces cerevisiae* strain INVSC1. Yeast strains were incubated in SG-Ura liquid medium at 30 °C on a shaker for 24 h to induce *AeIQM* gene expression. Subsequently, the optical density (OD_600_) of the yeast cultures was measured using a multifunctional enzyme marker (PerkinElmer EnSpire, Waltham, MA, USA), and the cultures were adjusted to an equal cell density. The yeast cells were then collected for stress assays. To examine the growth of yeast cells under stress conditions, the collected yeast cells were treated with 1.0 M NaCl for 6 h. A volume of 2.0 μL of yeast cells was spotted onto solid SG-Ura medium supplemented with 1.0 M NaCl, followed by incubation at 30 °C for 3–4 days using Memmert Incubator IN75. The growth of yeast cells was assessed based on the observed phenotypes. Furthermore, to evaluate the survival rates of yeast cells after salt stress, the cell densities were measured after incubation. The yeast cells were adjusted to an equal cell density and then cultured in 0.5 M, 1 M, and 2 M NaCl at 30 °C with shaking for 24 h. The cell densities were measured following each treatment.

### 2.8. Statistical Analysis

The data presented are the means of three replicates analyzed using Student’s *t*-test in Prism software (v9.5.0), with error bands indicating standard deviations. Asterisks above columns indicate significant differences between rows (* *p* < 0.05, ** *p* < 0.01, *** *p* < 0.001).

## 3. Results

### 3.1. Identification and Phylogenetic Analyses of IQM Gene Family in Kiwifruit

IQM proteins are plant-specific, calmodulin-binding proteins that are independent of calcium signaling and are characterized by the presence of a single IQ motif [16]. In this study, a comprehensive search was carried out using the known sequences of AtIQM and OsIQM proteins as a reference, and 10 AeIQM proteins containing characteristic IQ motifs were identified. The lengths of CDS ranged from 1026 to 1889 bps, and the encoded proteins exhibited diverse lengths, ranging from 341 to 632 amino acids, as recorded in Table 1. Accordingly, their predicted molecular weights ranged from 38.22 to 71.54 kDa. Interestingly, the majority of these proteins displayed theoretical isoelectric point (pI) values that fell within the alkaline range, ranging from 7.99 to 9.10. However, it is worth highlighting that AeIQM5 exhibited a slightly lower pI value of 6.98, while AeIQM6 and AeIQM9 demonstrated even lower pI values of 6.63 and 5.32, respectively.

**Figure 1:**
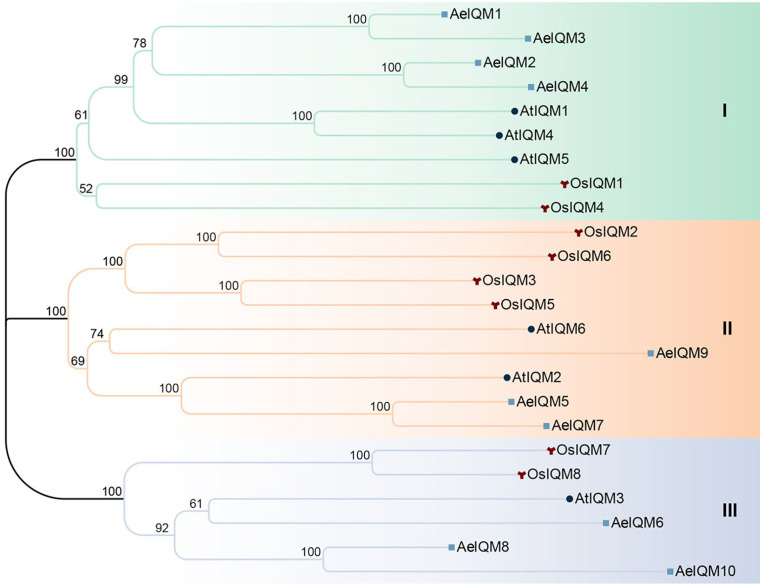
Phylogenetic analysis of proteins from *A. eriantha* (AeIQMs), *O. sativa* (OsIQMs), and *A. thaliana* (AtIQMs). The construction of the tree employed the neighbor-joining method with 1000 bootstrap replicates using MEGA11.0. Node reliability, based on 1000 bootstrap verifications, is represented by the numbers on the branches. Classification results are indicated by different colors: green—subfamily (**I**); orange—subfamily (**II**); blue—subfamily (**III**). To differentiate IQM proteins from the same species, distinctive geometric patterns were implemented: circle—*A. thaliana*; wye—*O. sativa*; square—*A. eriantha*.

To gain deeper insights into the evolutionary patterns and phylogenetic relationships, a phylogenetic tree was constructed for the IQM protein members derived from *A. eriantha*, *A. thaliana*, and *O. sativa*. The phylogenetic tree revealed the presence of three distinct subfamilies (I, II, and III) encompassing all the IQM members (Figure 1). Notably, subfamilies I and II emerged as the most prominent clades, each comprising nine IQMs. Collectively, these two subfamilies accounted for 37.5% of the total *IQM* genes under consideration.

### 3.2. Chromosomal Locations and Gene Duplication

**Figure 2:**
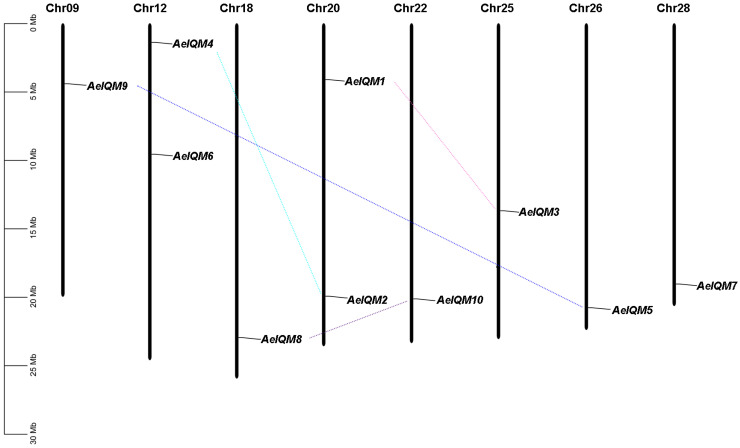
Chromosomal distribution and segmental duplication events of *IQM* genes in kiwifruit. Ten *AeIQM* genes are mapped to eight chromosomes. The duplicated paralogous pairs of *AeIQM* genes are connected with lines. Each chromosome is labeled with its respective numeric identifier displayed at the top. The length of each chromosome is represented on the left scale, measured in megabases (Mb).

To accurately determine the chromosomal orientation of the 10 *IQM* genes in kiwifruit, we constructed a collinearity map using location information obtained from the kiwifruit database (Figure 2). These *AeIQM* genes were mapped onto eight different chromosomes, namely, chromosomes 9, 12, 18, 20, 22, 25, 26, and 28. Notably, chromosomes 12 and 20 demonstrated the presence of two *IQM* genes each, while the remaining chromosomes encompassed one *IQM* gene each. This non-random pattern of *IQM* gene distribution aligns with the observations made in previous studies [21].

Gene duplication, which is considered a crucial mechanism driving biological evolution, occurs in three main ways: transposition, tandem duplication, and segmental duplication [42,43]. Notably, segmental duplication has been identified as a major driver for the amplification of numerous gene families [40,44,45]. In this study, we investigated gene duplication events to gain further insights into the expansion mechanisms of the IQM family in kiwifruit. A total of four segmental duplication events, *AeIQM1*/*AeIQM3*, *AeIQM2*/*AeIQM4*, *AeIQM7*/*AeIQM9*, and *AeIQM8*/*AeIQM10*, were detected in the kiwifruit genome based on the Ka/Ks analysis and the chromosomal distribution of the *AeIQM* genes (Figure 2, Table 2). Among the four *AeIQM* gene pairs, no pair of *AeIQM* genes was clustered on the same chromosome, suggesting that segmental duplication is the mechanism responsible for generating these duplicated gene pairs. Therefore, segmental duplication plays a significant role in the amplification and evolution of the *IQM* gene family in kiwifruit.

### 3.3. Gene Structure and Conserved Motifs of Kiwifruit IQM Proteins

**Figure 3:**
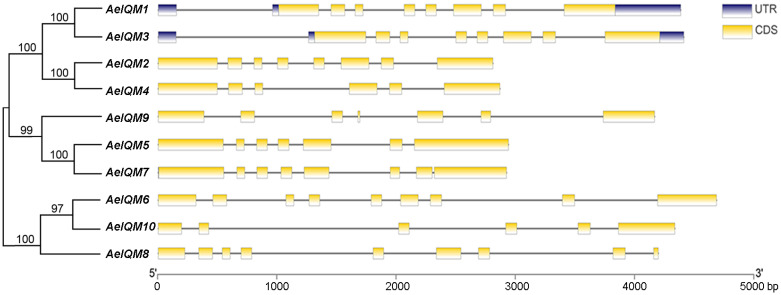
Phylogenetic relationships and gene structures of *AeIQM* genes. The unrooted phylogenetic tree of *AeIQM* proteins was constructed using the NJ method with 1000 bootstrap replicates. The CDS and untranslated regions (UTRs) are visually represented by yellow and blue boxes, respectively, while black lines indicate the introns. The scale provided at the bottom facilitates the estimation of the size of each *IQM* gene.

The diversity in gene structure serves as a fundamental aspect of the classification of gene families. To further investigate the gene features of *AeIQM* genes, an analysis of their exon/intron organization was performed (Figure 3). The structural diagram revealed that the majority of *AeIQM* genes exhibited variable numbers of exons, ranging from five to eight. Importantly, it should be noted that among the *IQM* gene family, genes 1 and 3 were observed to contain the UTR region, whereas the remaining genes lacked this feature. Moreover, within each subfamily, closely related *AeIQM* genes displayed consistent gene structures, either in terms of the number or length of introns/exons.

**Figure 4:**
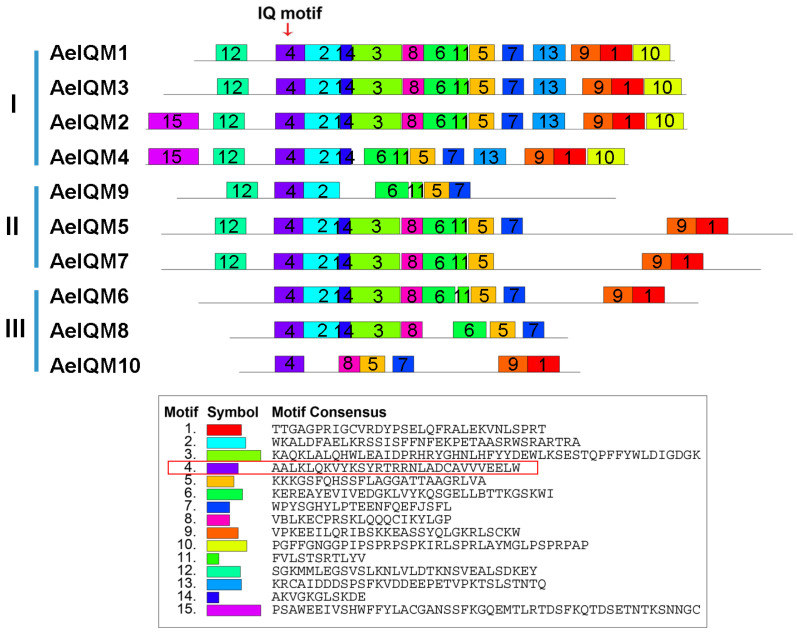
The distribution of motifs in AeIQM proteins. The identification of motifs was performed using the online MEME program. Each motif is denoted by a distinct colored box, with its assigned serial number positioned in the center of the box. The location of the IQ motif is indicated by an arrow positioned above the diagram, while the amino acid sequence of the IQ motif is depicted in the red box in the diagram.

The conserved motifs of 10 AeIQM proteins and a total of 15 potentially conserved motifs, named motifs 1–15 (Figure 4), were examined. Motif 4, after being subjected to a comprehensive search of the Pfamscan and SMART databases, was annotated to encode the IQ motif. It is worth noting that all AeIQM proteins were found to possess motifs 4, 5, and 7. Among the identified motifs, AeIQM2 exhibited the highest number, encompassing motifs 1–15, while AeIQM10 displayed the smallest number of motifs, consisting of only 6 motifs (motifs 1, 4, 5, 7, 8, and 9). Furthermore, certain motifs were found to be specific to particular subfamilies. For instance, motifs 10 and 13 were uniquely present in subfamily I, while motif 12 was absent in subfamily II. Moreover, motif 15 was exclusively observed in AeIQM2 and AeIQM4. These distinct motifs may contribute to functional differentiation among the IQM proteins in kiwifruit.

### 3.4. Analysis of Cis-Acting Elements of AeIQM Gene Promoters

**Figure 5:**
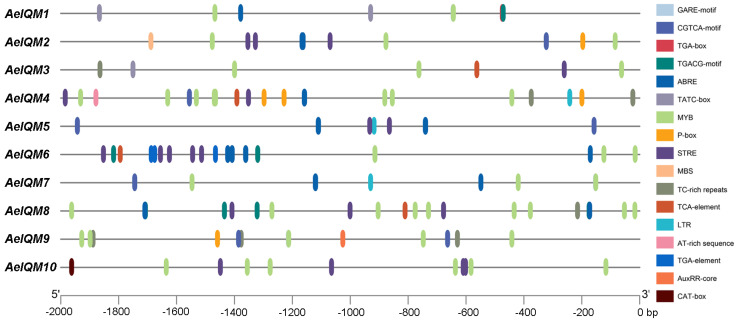
The *cis*-acting elements in the promoter sequences located 2000 bp upstream of *AeIQM* genes, which were predicted using PlantCARE. Distinct cis-acting elements are visualized through the utilization of various colored rectangles.

The role of *cis*-acting elements in gene promoters, including their type and number, is widely recognized in determining gene function. In this study, the promoter of the *AeIQM* gene in kiwifruit was analyzed. The results showed that the *AeIQM* gene promoter contains six different classes of *cis*-acting elements associated with hormonal and stress responses (Figure 5). Figure 5 displays significant variations in the composition and abundance of response elements among the *AeIQM* genes. Among these elements, defense and stress-responsive elements (LTR, TC-rich repeats, MYB, MBS, and STRE) were the most prevalent, with a total of 75 occurrences. These were followed by MeJA-responsive elements (CGTCA-motif and TGACG-motif), of which there were 28 occurrences. ABA response elements (ABRE) were identified in 25 occurrences, while gibberellin-responsive elements (TATC-box, P-box, and GARE-motif) were observed in 13 occurrences. Auxin-responsive elements (AuxRR-core and TGA-element) were present in five occurrences, whereas salicylic-acid-responsive elements (TCA-element) were found in four occurrences. Furthermore, the number of other *cis*-elements present in *AeIQM* genes varied from 7 to 24, with *AeIQM4*, *AeIQM6*, and *AeIQM8* having 24 *cis*-elements, while *AeIQM3* possessed a minimum of 7 elements. Consequently, different members within the *AeIQM* gene family may exhibit distinct functions in mediating kiwifruit’s responses to both hormones and abiotic stresses.

### 3.5. Effects of Abiotic Stress on AeIQM Gene Expression

**Figure 6:**
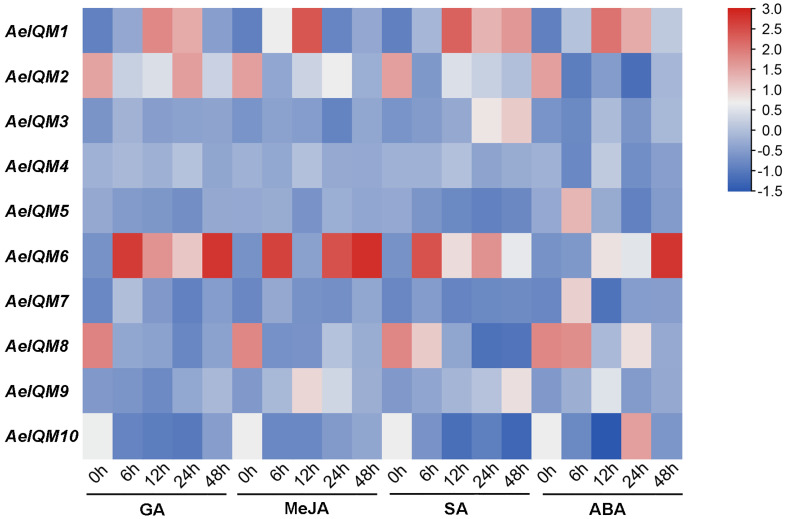
Expression profile of *AeIQM* genes under different hormone treatments. The expression levels of *AeIQM* genes in leaf tissues under different hormone treatments were analyzed via real-time quantitative PCR, with three biological and technical replicates. The resulting data were visualized in a heat map format using TBtools software. The color bar located to the right of the Figure represents the relative signal intensity values.

Based on the above analysis of *cis*-acting elements in promoters, it is likely that the regulation of *IQM* genes in kiwifruit is influenced by hormonal stimuli and abiotic stresses. To further confirm the functionality of the *AeIQMs*, a series of experiments involving various hormonal treatments (GA, MeJA, SA, and ABA), as well as salt and drought stresses, was conducted on *A. eriantha*. As depicted in Figure 6, analysis of gene expression profiling revealed that most *AeIQM* genes were prompted by hormonal treatments. Specifically, after GA treatment, *AeIQM1*, *AeIQM2*, *AeIQM4*, and *AeIQM6* exhibited high expression levels, while five *AeIQM* genes (*AeIQM1*, *2*, *4*, *6*, *9*) were upregulated under JA stress. Apart from *AeIQM6* and *AeIQM10*, the other eight *AeIQM* genes demonstrated relatively high expression levels following SA stress. Additionally, all *AeIQM* genes demonstrated responsiveness to ABA stress. Furthermore, certain differences were observed among these genes. For instance, *AeIQM1* and *AeIQM6* displayed strong expression levels under each hormone treatment. *AeIQM3* exhibited an increase in expression only under SA and ABA stress, while *AeIQM5’s* expression level increased solely under ABA stress.

**Figure 7:**
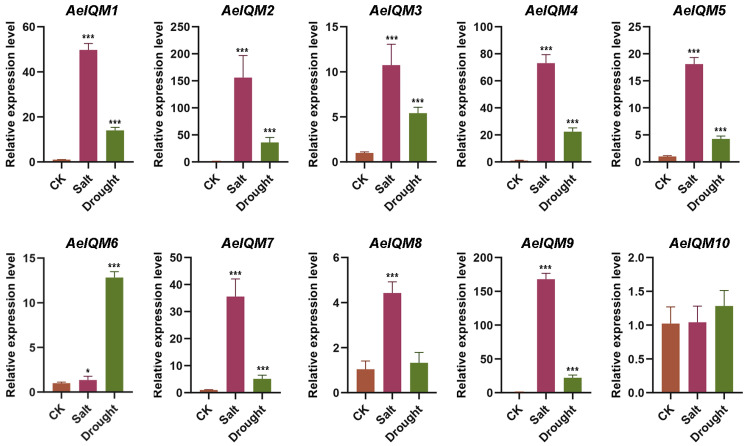
Expression levels of *AeIQM* genes under salt and drought stress. Three biological and technical replicates were used to calculate the error bars. Asterisks are used to indicate significant upregulation of the corresponding genes as determined by a *t*-test analysis (* *p* < 0.05, *** *p* < 0.001).

Except for *AeIQM10*, all genes demonstrated significant upregulation under salt stress (Figure 7). Likewise, the gene expression patterns observed under drought stress resembled those obtained from salt treatment, except for *AeIQM8* (Figure 7). However, it is noteworthy that salt treatment elicited more pronounced alterations in gene transcripts compared to drought treatment. These findings suggest a crucial involvement of specific *AeIQM* genes in governing the responses to ABA, IAA, MeJA, and high salinity stress in kiwifruit.

### 3.6. Salt Stress Resistance Analysis of AeIQM Genes in Yeast

**Figure 8:**
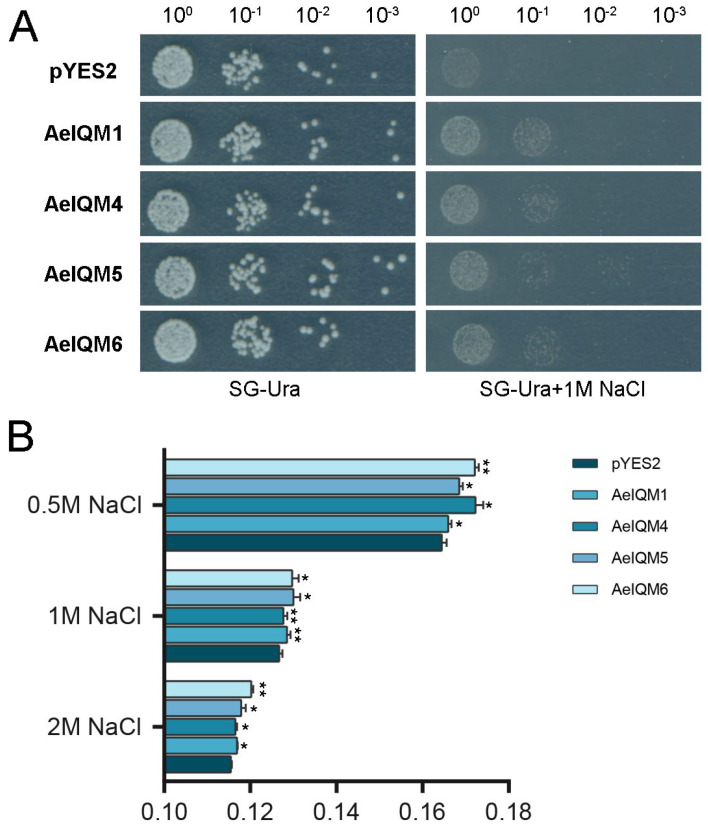
The growth activity of INVSC1 (pYES2) and INVSC1(pYES2-AeIQM) under salt treatment. (**A**) The growth of yeast cells under stress conditions. The yeast cells were adjusted to an equal cell density and then treated with 2.0 M NaCl for 6 h. A volume of 2.0 μL of yeast cells was spotted onto solid SG-Ura medium supplemented with 2.0 M NaCl, followed by incubation at 30 °C for 3 days. The growth of yeast cells was assessed based on the observed phenotypes. (**B**) The survival rates of yeast cells after salt stress. The yeast cells were adjusted to an equal cell density and then cultured in 0.5 M, 1 M, and 2 M NaCl at 30 °C with shaking for 24 h. The cell densities were measured following each treatment. Asterisks were used to indicate significant upregulation of the corresponding genes as determined by a *t*-test analysis (* *p* < 0.05, ** *p* < 0.01).

To investigate the impact of salt stress on pYES2-AeIQMs yeast cell growth, two groups of yeast cells were assessed: one group harboring empty pYES2 plasmids and the other group harboring pYES2-AeIQM plasmids. Following treatment with NaCl, the growth performance of these yeast cells was evaluated. The findings demonstrated that, under normal conditions, all yeast cells exhibited similar growth rates and thrived well. However, notable differences were observed after NaCl treatment (Figure 8A). In the presence of NaCl, yeast cells transformed with AeIQMs exhibited significantly enhanced growth compared to the control group. Furthermore, to evaluate the survival rates of yeast cells after salt stress, the yeast cells were adjusted to an equal cell density and then cultured in 0.5 M, 1 M, and 2 M NaCl at 30 °C with shaking for 24 h. The results revealed that the amount of yeast decreased with increasing salt concentration. However, it was observed that the number of yeast cells expressing AeIQMs was significantly higher than the control group (Figure 8B). These results imply that AeIQMs confer salinity tolerance to transgenic yeast cells, thereby suggesting their involvement in salinity tolerance mechanisms.

## 4. Discussion

During the process of evolution, plants have developed diverse physiological mechanisms to effectively combat stress. Notably, previous investigations have highlighted the indispensable role of calcium ions (Ca^2+^) as a pivotal second messenger in mediating the responses of plants to various environmental stimuli [12,46,47,48]. As key components of plant-specific calcium signaling pathways, *IQM* genes are believed to play a crucial role in mediating the crosstalk between multiple signaling pathways in the context of plant growth [23,24]. While studies exploring the *IQM* gene family have been conducted in some species, including rice [22], *Arabidopsis* [16], and soybean [21], there is currently a dearth of published research about the *IQM* gene family in kiwifruit. In this study, a comprehensive analysis was conducted to identify and investigate the *IQM* gene family in kiwifruit. A total of 10 *IQM* genes were identified, and subsequent bioinformatics approaches were employed to analyze these genes at the whole-genome level. The members of the *AeIQM* gene family displayed a range of encoded protein lengths, varying from 341 to 632 amino acids. The MW of these proteins was within the range of 38.22 to 71.54 kDa. Interestingly, the majority of the proteins exhibited high theoretical pI, ranging from 7.99 to 9.1. Based on their phylogenetic relationships with IQM proteins from *O. sativa* and *Arabidopsis,* these *AeIQM* genes were categorized into three distinct subfamilies, namely, subfamily I, subfamily II, and subfamily III. Notably, subfamilies I and II contained the most members, with each subfamily comprising nine IQMs, accounting for 37.5% of the total IQM proteins count in kiwifruit, a finding that is consistent with previous reports in other plant species [16,21,22]. Members within the same subfamily exhibited close similarity in terms of phylogenetic relationships (Figure 1), exon–intron structure (Figure 3), and motif distribution (Figure 4), thereby supporting the reliability of the subfamily classification. These results suggest that the AeIQM protein family has undergone relatively conserved evolution and that IQMs within each subfamily may share similar functions. In our study, by comparing the IQM protein sequences of multiple species, the IQM proteins from three distinct subfamilies were further divided into two groups: dicots (*Arabidopsis*, kiwifruit) and monocots (rice). In addition, 10 AeIQMs from kiwifruit were clustered in the same clade as *Arabidopsis*, indicating that AeIQM and AtIQM are more closely related than OsIQMs from rice. This also indicates the sequence conservation and significant divergence in monocots and dicots.

In comparison to *Arabidopsis*, which possesses six members of the *IQM* gene family, kiwifruit exhibits a significantly larger *IQM* gene family with a total of ten members. This increase in gene number is a pattern that has already been reported in numerous gene families in kiwifruit. For instance, in *A. eriantha*, the gene family of growth-regulating factors (*GRFs*) comprises 26 members, surpassing the 9 members found in *Arabidopsis* [45]. Similarly, the gene family of heat shock transcription factors (*HSFs*) consists of 20 members in *Arabidopsis* and 41 members in kiwifruit [40]. The phenomenon of expanded gene numbers in kiwifruit can be attributed to genome-wide duplication (WGD) events that have occurred in its evolutionary history. WGD, which is a common occurrence in plants, leads to the duplication of gene copies within the genome. Kiwifruit has undergone at least two WGD events, estimated to have taken place approximately 27 and 80 million years ago, leading to the establishment of the duplicated genome [49,50]. The kiwifruit genome has likely experienced a greater expansion of *IQM* genes in comparison to other species. Our analysis reveals the presence of four paralogous pairs among the ten *AeIQM* genes, all of which arose from segmental duplication rather than tandem duplication (Table 2). The identified results provide evidence suggesting that segmental duplication has played a prominent role in the extensive evolutionary history of the *IQM* gene family in kiwifruit. Notably, analysis of the Ka/Ks ratio for all four identified pairs of duplicated genes demonstrated values below 1 (Table 2), indicating the prevalence of purifying selection acting upon these gene pairs [51].

The presence of the IQ motif is crucial for the functional involvement of *IQM* genes in calcium-signaling processes [23]. Through the utilization of MEME analysis in our study, it was revealed that motif 4, motif 5, and motif 7 were consistently observed across almost all members of the *AeIQM* gene family, with motif 4 notably containing the IQ motif. Notably, there were variations in the number and distribution of motifs among different AeIQM proteins. For instance, AeIQM2 exhibited the highest number of motifs (motifs 1–15), while AeIQM10 displayed the fewest motifs, comprising only 6 motifs. Furthermore, specific motifs were observed to be exclusive to certain subfamilies. For example, motifs 10 and 13 were identified solely within subfamily I, while motif 12 was absent in subfamily II. Additionally, motif 15 was uniquely present in AeIQM2 and AeIQM4. These observations regarding variations in motif number and distribution among the proteins align with previous research findings [16]. Consequently, it can be postulated that these divergent motifs may contribute to functional divergence among the IQM proteins in kiwifruit.

Previous studies on *Arabidopsis* have revealed diverse functionalities of *AtIQM* genes. For instance, *AtIQM1* has been demonstrated to regulate stomatal movement by modulating reactive oxygen species (ROS) levels, and it also contributes to plant disease response signaling through the promotion of JA synthesis [23,24]. In contrast, *AtIQM4* plays a role in positively regulating seed dormancy and germination by modulating ABA levels [25]. Additionally, *AtIQM5* is involved in the regulation of flowering by modulating the transition from the juvenile to the adult phase. Furthermore, it interacts with IAAs, which are key auxin signaling repressors, to promote lateral root and callus formation [26]. However, the functional characterization of *IQM* genes in kiwifruit remains unexplored, as their specific roles and activities have not yet been elucidated. In the current investigation, we sought to elucidate the functional characteristics of the *AeIQM* gene promoters by assessing the presence of different types and numbers of *cis*-acting elements. Phytohormones exert a prominent influence on plant growth regulation and the adaptation of plants to diverse abiotic stresses [7,52,53]. The promoter’s analysis revealed that the *AeIQM* gene promoter encompasses six distinct categories of *cis*-acting elements, including IAA, ABA, SA, MeJA, GA, and defense and stress-responsive elements. To further confirm the function of *AeIQM* genes, we subjected kiwifruit to different hormone stress, salt stress, and drought stress factors. Consequently, all *AeIQM* genes exhibited significant increases in expression levels in response to hormonal treatments. Particularly, following the application of GA treatment, four *AeIQM* genes displayed elevated expression levels, whereas, under JA stress, five *AeIQM* genes were upregulated. Moreover, it was observed that all *AeIQM* genes possessed the capacity to respond to ABA stress. Except for *AeIQM10*, all genes demonstrated significant upregulation under salt stress. Likewise, the gene expression patterns observed under drought stress resembled those obtained from salt treatment, except for *AeIQM8*. However, it is noteworthy that salt treatment elicited more pronounced alterations in gene transcripts compared to drought treatment. These findings suggest a crucial involvement of specific *AeIQM* genes in governing the responses to ABA, IAA, MeJA, and high salinity stress in kiwifruit. Consequently, the various members within the *AeIQM* gene family may fulfill unique roles in mediating kiwifruit’s responses to abiotic stresses.

Soil salinization, characterized as a highly detrimental abiotic factor, exerts adverse effects on seed germination, crop growth, and overall productivity [27,31,54]. Salinity stress has proven to be a notable and influential factor that substantially affects kiwifruit quality and yield [27]. The *IQM* gene family assumes crucial regulatory functions across various aspects of plant growth and adaptation to abiotic stressors. Nevertheless, comprehensive exploration of the *IQM* gene family’s involvement in kiwifruit’s response to diverse abiotic stresses, particularly salt stress, remains limited. In this study, the data presented in Figure 7 demonstrate the *IQM* genes can be induced by salt stress, suggesting their potential participation in salt stress response. To further investigate their role in salt stress, we conducted stress experiments in yeast. Under normal conditions, all yeast cells exhibited similar growth rates and thrived well. However, notable differences were observed following exposure to NaCl treatment, as depicted in Figure 8. These findings imply that AeIQMs confer salinity tolerance to transgenic yeast cells, thereby implicating their involvement in mechanisms associated with salinity tolerance.

## 5. Conclusions

In conclusion, a total of ten *AeIQM* genes have been successfully identified in kiwifruit and classified into three different subfamilies (I–III). The *AeIQM* genes are distributed on eight chromosomes, of which four pairs of *AeIQM* gene duplications are associated with segmental repeat events. These genes exhibit diverse structural features and encompass a range of functions. Specifically, their expression patterns under various hormone treatments indicate that most *AeIQM* genes can be induced by abiotic stresses such as salt and drought. Furthermore, overexpression of *AeIQM* in yeast significantly enhances the salt tolerance of yeast. Therefore, the study of *AeIQM* is considered to be of great significance. The comprehensive analysis of the kiwifruit *IQM* gene family conducted herein establishes a solid theoretical basis.

## Figures and Tables

**Table 1:** The information on *AeIQM* genes identified in kiwifruit (*A. eriantha*).

Name	Gene ID	Chr.	Location Coordinates	CDS Length (bp)	Protein Length (aa)	Molecular Weight (kDa)	pI
*AeIQM1*	DTZ79_20g03230	Chr20	4097436–4101769 (−)	1446	481	53.96	8.57
*AeIQM2*	DTZ79_20g13100	Chr20	19923399–19926174 (+)	1644	547	61.58	8.39
*AeIQM3*	DTZ79_25g04840	Chr25	13677555–13681914 (−)	1572	523	58.30	8.70
*AeIQM4*	DTZ79_12g01010	Chr12	1370000–1372833 (−)	1467	488	54.24	9.10
*AeIQM5*	DTZ79_26g12800	Chr26	20764403–20767307 (+)	1899	632	71.54	6.98
*AeIQM6*	DTZ79_12g06920	Chr12	9544437–9549067 (−)	1503	500	56.05	6.63
*AeIQM7*	DTZ79_28g12820	Chr28	19031981–19034869 (+)	1806	601	68.15	8.71
*AeIQM8*	DTZ79_18g12720	Chr18	22944547–22948694 (+)	1017	338	39.15	7.99
*AeIQM9*	DTZ79_09g03830	Chr09	4394286–4398402 (−)	1320	439	50.55	5.32
*AeIQM10*	DTZ79_22g10670	Chr22	20117392–20121676 (+)	1026	341	38.22	8.98

**Table 2:** The divergence between paralogous *IQM* gene pairs in kiwifruit.

NO.	Paralogous Pairs	Ka	Ks	Ka/Ks	Duplicate Type
1	*AeIQM1–AeIQM3*	0.081680638	0.209879171	0.389179342	Segmental duplication
2	*AeIQM2–AeIQM4*	0.051740668	0.152338558	0.339642625	Segmental duplication
3	*AeIQM7–AeIQM9*	0.412184526	1.815557259	0.227029208	Segmental duplication
4	*AeIQM8–AeIQM10*	0.152649613	0.210881697	0.723863739	Segmental duplication

## Data Availability

All data are displayed in the manuscript and Supplementary File.

## Supplementary Materials

The following supporting information can be downloaded at: https://www.mdpi.com/article/10.3390/genes15020147/s1, Table S1: All primers used for qRT-PCR and vector construction.
