# Supplementary material for: Genome-Wide Identification of the IQM Gene Family and Their Transcriptional Responses to Abiotic Stresses in Kiwifruit (Actinidia eriantha)
Source: Genes (Basel). 2024 Jan 23;15(2):147. doi: 10.3390/genes15020147 (PMC10887524; doi:10.3390/genes15020147)
Supplement: Supplementary file 1 [file genes-15-00147-s001.zip › genes-2830420-supplementary.pdf]

Supplementary Materials

Genome-Wide Identification of the *IQM* Gene Family and Their Transcriptional Responses to Abiotic Stresses in Kiwifruit

Minyan Xu 1, Zhi Zhang 1, Chengcheng Ling 1,2, Yuhuan Jiao 1 and Xin Zhang 1,*
1 National Engineering Laboratory of Crop Stress Resistance Breeding, School of Life Sciences, Anhui Agricultural University, Hefei 230036, China
2 College of Food and Bioengineering, Bengbu University, Bengbu 233030, China
* Correspondence: xinzhang@ahau.edu.cn

Table S1. All primers used for qRT-PCR and vector construction.

Primer name	Sequence (5' to 3')	Tm (℃)	Amplification temperature (℃)	Final concentration (μM)
AeActin1-qF	CTTACAGAGGCACCACTCAACC	58	60	0.25
AeActin1-qR	ATAGATGGGGACCGTGTGACT	58	60	0.25
AeIQM1-qF	TTGATGGACCTACCCACG	56	60	0.25
AeIQM1-qR	CCCTTACACACCCGATGCGT	63	60	0.25
AeIQM2-qF	ACGTGGAAGCCTCAGTGTTT	59	60	0.25
AeIQM2-qR	TCGAGTGCCCGGAATTGTAG	59	60	0.25
AeIQM3-qF	CTGAAGCGCCTGTGTTTGAC	58	60	0.25
AeIQM3-qR	GTAGTTCCTCACGCACCCAA	58	60	0.25
AeIQM4-qF	ACACGAAGAAACCTTGCGGA	59	60	0.25
AeIQM4-qR	TTTCCCACTTTGGCAGCTCT	59	60	0.25
AeIQM5-qF	AGCATTCGAGCTTCTTGGCT	59	60	0.25
AeIQM5-qR	GACCGCCTTTAGGATGCCTT	59	60	0.25
AeIQM6-qF	CCGAGAACTCCTCCAACACC	59	60	0.25
AeIQM6-qR	GAGATCCGACGAAGGTGGTG	59	60	0.25
AeIQM7-qF	GAAGCTGTTGGACTTTGCCG	59	60	0.25
AeIQM7-qR	CGTGAAATTGCGGTCTCGTG	59	60	0.25
AeIQM8-qF	ACGCATCGCAATCACAACAG	59	60	0.25
AeIQM8-qR	CCCCAGTTTGTTTGTGCACC	60	60	0.25
AeIQM9-qF	GACACGAGGATTATTAGCGATTCT	57	60	0.25
AeIQM9-qR	GGGCTTTTATTGTGGTCTTTTAGG	57	60	0.25
AeIQM10-qF	AAGAGTGCCCGAGATCATGG	59	60	0.25
AeIQM10-qR	CCATAAGTCGTCCAGCAGCT	58	60	0.25
AeIQM1-pYES2NB-F	cgataaggtacctaaggatccATGGGTTTTTGGAAAGAAGATTCC	68.2	58	0.25
AeIQM1-pYES2NB-R	tgctggatatctgcagaattcATGAACAGTTGAAGCAGCAACCT	67.6	58	0.25
AeIQM4-pYES2NB-F	cgataaggtacctaaggatccATGGGCTTATCCCTATCCTTACTTC	69.6	58	0.25
AeIQM4-pYES2NB-R	tgctggatatctgcagaattcGCCAGAGGTGGGGATGGG	71	58	0.25
AeIQM5-pYES2NB-F	cgataaggtacctaaggatccATGGGGATATCCTTTTCGTGC	68.4	58	0.25
AeIQM5-pYES2NB-R	tgctggatatctgcagaattcCCGTATTCTCGTGGGGTCATG	69.4	58	0.25
AeIQM6-pYES2NB-F	cgataaggtacctaaggatccATGGAGATCGAGACTCAAACGC	69.9	58	0.25
AeIQM6-pYES2NB-R	tgctggatatctgcagaattcGACAGCAAACGACGACTGGC	69.9	58	0.25
